# The interaction between frailty and patient characteristics and its association with hospital-related adverse events in older adults

**DOI:** 10.1186/s12877-026-07854-4

**Published:** 2026-06-23

**Authors:** Faris Alotaibi, Bradley Manktelow, Abdullah Alshibani, Adam Linton, Jay Banerjee

**Affiliations:** 1 Emergency Medical Care Department, College of Applied Medical Sciences, Imam Abdulrahman Bin Faisal University, Dammam, Saudi Arabia; 2 School of Healthcare, College of Life Sciences, University of Leicester, Leicester, UK; 3 Emergency Medical Services Department, College of Applied Medical Sciences, King Saud Bin Abdulaziz University for Health Sciences, Riyadh, Saudi Arabia; 4 King Abdullah International Medical Research Center, Riyadh, Saudi Arabia; 5 Department of Emergency and Specialist Medicine (ESM), University Hospitals of Leicester NHS Trust, Leicester, UK

**Keywords:** Frailty, Adverse events, Patient safety, Clinical frailty score

## Abstract

**Background:**

Frailty is a state of increased vulnerability to stressors that predisposes individuals to adverse outcomes. Hospital-related adverse events (AEs) are complications not directly caused by patients’ pre-existing conditions. While age has been widely studied, less is known about the effect of frailty and its interaction with patient demographic and clinical characteristics on the risk of having at least one adverse event (AE) among acutely admitted older adults.

**Methods:**

In this study, AEs were operationally defined as Datix-reported patient safety incidents with the potential to cause harm. Poisson regression models were used to estimate Relative Risk (RRs) and 95% Confidence Intervals (CIs) for the association between frailty and the likelihood of experiencing at least one AE during admission. Frailty was assessed at the Emergency Department (ED) using the Clinical Frailty Scale (CFS) and modelled as the primary predictor. Individual models were then used to assess crude associations for age, gender, ethnicity, ED wait time, and In-patient (IP) Length of Stay (LOS). Interaction terms between frailty and each characteristic were tested using likelihood ratio tests. Backward stepwise elimination was used to obtain a multivariable model retaining only variables that significantly improved model fit (*p* < 0.01).

**Results:**

A total of 158,470 hospital admissions with a recorded frailty score were included. Statistically significant interactions were found between frailty and age, ED wait time, and IP LOS in relation to the risk of experiencing at least one AE (all *p* < 0.01). Multivariable modelling showed that the interaction between frailty and IP LOS was the only interaction that significantly improved model fit.

**Conclusion:**

The risk of AEs was associated with increasing frailty, and this association varied with IP LOS. Frailty assessment may help identify patients at increased risk of Datix-reported patient safety incidents, although the observed interaction with IP LOS does not imply causation.

**Supplementary Information:**

The online version contains supplementary material available at https://doi.org/10.1186/s12877-026-07854-4.

## Key points

• Frailty was independently associated with increased risk of adverse events during acute hospital admission.

• The association between frailty and the risk of adverse events differed by age, emergency department wait time, and inpatient length of stay.

• A significant frailty–length of stay interaction indicated that the association between frailty and adverse events risk varied according to inpatient length of stay.

• Interactions between frailty and gender or ethnicity were not statistically significant.

• In the final multivariable model, the frailty–length of stay interaction was the only interaction that significantly improved model fit.

**Supplementary Information:**

The online version contains supplementary material available at https://doi.org/10.1186/s12877-026-07854-4.

## Background

The number of people aged 65 and older is growing in the UK. In 2020, 18.6% of the UK population was aged 65 or over [1]. This proportion is projected to reach 24% in 2038, which is associated with increased health and social care needs [2, 3]. As people age, the prevalence of age-related syndromes, e.g. frailty, increases. Approximately one in ten individuals aged 65 and older live with frailty, with a higher proportion of females affected compared to males [4]. 

Frailty is an age-related syndrome that affects older people and is characterised by a decline in the physiologic function of many body systems [5]. It is described as a state of increased vulnerability and inability to maintain homeostasis after a stressor, which may cause adverse outcomes, like falls, delirium, disability and abrupt changes in health status [5–8]. Various methods are used to assess frailty, including Fried’s phenotype model [5], the Frailty Index based on cumulative deficits [9], and the Clinical Frailty Scale (CFS) [10]. In its most updated form, the CFS is a nine-point clinician-rated scale that captures both functional and cognitive status [11]. As frailty is associated with the risk of adverse outcomes, the risk of hospital related adverse events (AEs) also increase for this population when receiving care.

An adverse event (AE) is any injury occurred during a healthcare episode that is not primarily related to the patient illness, comorbidities or past medical history [12]. Globally, studies estimate that approximately 1 in 20 patients may experience an AE during a hospital admission [13]. and 50% of AEs are possibly preventable [14]. AEs could lead to a prolonged hospital stay, disability, or death [15]. There are many methods to report and identify AEs in hospitalised patients, including voluntary incident reporting, chart review, or patient interviews [16]. 

While previous studies have demonstrated associations between frailty and adverse outcomes, less is known about how the degree of frailty interacts with patient demographics (age, ethnicity, and gender) and clinical characteristics (length of stay and wait times) to influence the risk of AEs in acutely admitted older patients. This study adds to the existing literature by using a large routinely collected hospital dataset and frailty assessment using the CFS at emergency department (ED) presentation to examine the association between frailty and the risk of experiencing at least one AE during hospital admission, including whether this association varies through interactions between frailty and patient demographic and clinical characteristics in older acutely admitted patients.

## Methods

### Setting

This study was undertaken at a single-centre university teaching hospital in the East Midlands of the United Kingdom. It provides emergency care to 240,000 attendees annually, including 50,000 older people aged ≥ 65 at its ED. The hospital serves a local population of 1.1 million residents [17]. Routine frailty assessment using the CFS was introduced into the electronic health record (EHR) in October 2017 for all patients aged 65 years and older presenting to the ED.

### Study type and sample

We performed a retrospective observational study of patients aged ≥ 65 who presented acutely to the ED or were admitted from the ED to in-patient (IP) care. Frailty assessment was performed at the time of ED presentation using the CFS and was not reassessed again during hospitalisation. The degree of frailty was grouped as non-frail (CFS 1–3) and further stratified into mild (CFS 4–5), moderate (CFS 6), and severe (CFS 7–9) frailty. Only admissions with a documented frailty score were eligible for inclusion. To assess potential selection bias related to missing frailty data, a comparative analysis was performed between admissions with and without a recorded CFS among patients aged ≥ 65 years acutely admitted. Patient characteristics compared included age, gender, ethnicity, ED waiting time, IP admission status, length of stay (LOS), discharge destination, and in-hospital mortality.

In this study, AEs were operationally defined as Datix-reported patient safety incidents with the potential to cause harm. AEs were obtained from Datix, a national NHS web-based incident reporting system used to record and investigate patient safety incidents [18]. Although Datix captures a broad range of incidents, there is variation in what hospitals choose to record as AEs. This study focused on selected categories reported in Datix that are clinically relevant and likely to be associated with patient harm: pressure ulcers, moisture-associated skin damage, falls, hospital injury or trauma, infection-related incidents, medication errors, adverse drug reactions, self-harm, patient abuse, and incidents occurring during patient discharge or transfer. To support analysis, individual Datix-reported incidents were grouped into these broader AE categories. The specific Datix terms included within each category are detailed in Supplementary Table 1. The frequency of admissions with at least one AE by category across frailty groups is presented in Supplementary Table 2.

### Statistical analysis

Baseline characteristics were summarised across the four predefined frailty categories; the number of admissions and percentages was relative to the total included sample. Patient’s demographics variables (age, gender, ethnicity), ED measures (wait time in minutes, ED admission indicator), IP measures (LOS in nights, discharge destination), and in-hospital mortality was summarised across the four frailty categories. ED wait time and IP LOS were categorised to improve clinical interpretability and allow for potentially non-linear relationships with AE risk. ED wait time was defined as the duration of stay in the ED (minutes), while IP LOS was defined as the duration of the inpatient hospital stay (nights), as recorded in the hospital dataset. Continuous data were summarised as mean and standard deviation (SD) or median and interquartile range (IQR) as appropriate based on the normality of data distribution. Categorical data were presented as counts and percentages of the total admissions within each frailty category.

A series of Poisson regression models was applied to estimate the relative risk (RR) and 95% confidence intervals (CIs) of experiencing at least one AE during an acute admission. Robust standard errors were applied to account for potential variance misspecification and to ensure valid inference. A significance level of *p* < 0.01 was chosen to minimise the likelihood of false-positive findings due to multiple testing and to ensure that identified associations are not only statistically significant but also clinically meaningful, given the relatively large sample size. These models examined the association between frailty and key patient demographic and clinical characteristics specifically age, gender, ethnicity, ED wait time, and IP LOS, with the risk of experiencing at least one AE.

The significance of each interaction of patient’s characteristics with frailty was tested using likelihood ratio test comparing the interaction model with the main-effects-only model. Where the interaction was not statistically significant, the main effects of that characteristic adjusted for frailty were reported.

To enhance the model and identify the most meaningful interactions, a backwards stepwise elimination approach was used to select statistically significant variables and interactions for the multivariable model. At each step, the reduced model was compared with the previous using the Likelihood Ratio Test. The least statistically significant term was removed, while retaining the main effect of any variable involved in a remaining interaction. This process was repeated until only terms that significantly improved model fit remained (*p* < 0.01). A sensitivity analysis was subsequently performed by re-estimating the final retained multivariable model using patient-level clustered robust standard errors to account for within-patient correlation, as some patients contributed more than one admission during the study period. The dependent variable in all models was the occurrence of at least one AE during the hospital admission as defined previously. IP LOS was included as a representative measure of duration of exposure to the hospital environment. Collinearity diagnostics were also performed using the corresponding multivariable model without interaction terms to assess collinearity among the main predictors.

## Results

158,470 acute admissions with a frailty score recorded in the ED were analysed, drawn from 369,798 admissions between October 2017 and October 2023 among patients aged ≥ 65 years. Of the total admissions, 88,369 records had a missing ED data were excluded, since frailty assessment was not reported. Among admissions with available ED data, 158,470 (56.3%) had a recorded CFS score, while 122,959 (43.7%) had no documented frailty assessment. Admissions with a recorded CFS tended to involve older patients (median age 80 vs. 76 years), longer ED waiting times, higher admission rates, longer inpatient stays, and greater in-hospital mortality. Differences in ethnic distribution were modest, and gender distribution was similar between groups. See Table 1.

**Table 1:** Descriptive comparison between admissions with and without a reported CFS

Characteristic	No Frailty Score Reported	Frailty Reported
Total Admissions N (%)	122,959 (43.7%)	158,470 (56.3%)
Median Age (IQR)	76 (70–83)	80 (74–87)
Female N (%)	65,838 (53.5%)	85,906 (54.2%)
White Ethnicity N (%)	95,420 (77.6%)	132,646 (83.7%)
Median ED Wait Time in Minutes (IQR)	233 (134–397)	448 (272–732)
Admitted from ED to IP	51,155 (41.6%)	113,000 (71.31%)
Median IP LOS (IQR)	4 (1–10)	6 (2–12)
In-hospital Mortality N (%)	3337 (2.71%)	8875 (5.6%)
Discharge to a destination other than Home N (%)	7449 (14.56%)	20,196 (17.87%)

*ED* Emergency Department, *IP* Inpatient, *LOS* Length of Stay, *IQR* Interquartile Range

In the included 158,470 admissions with a recorded frailty score, the median age was associated with increasing frailty ranging from 74 years (IQR 69–80) in non-frail patients to 84 years (IQR 77–90) in those with severe frailty, and the proportion of female patients rose from 48.9% in those with no frailty to 58.4% in severe frailty. Across frailty groups, most patients were of White ethnicity peaking at 85% in moderate frailty category, followed by Asian ethnicity peaking at 13% in mild frailty, with other ethnic groups each accounting for < 2% across all frailty categories. See Table 2.

**Table 2:** Patients characteristics per frailty level

Patient characteristics	Non-Frail CFS 1–3	Mild Frailty CFS 4–5	Moderate Frailty CFS 6	Severe Frailty CFS 7–9
Total Admission. N (%)	35,691 (22.5%)	62,581 (39.5%)	37,068 (23.4%)	23,130 (14.6%)
Median Age (IQR)	74 (69–80)	80 (74–86)	84 (78–89)	84 (77–90)
Female % (N)	48.9% (*n* = 17,455)	53.2% (*n* = 33,333)	58.2% (*n* = 21,599)	58.4% (*n* = 13,519)
White Ethnicity % (N)	82.7% (*n* = 29,536)	83.3% (*n* = 52,177)	85.1% (*n* = 31,551)	83.8% (*n* = 19,382)
Median ED Wait Time in Minutes (IQR)	334 (202–556)	475 (293–771)	516 (326–815)	460 (292–730)
Median IP LOS in Nights (IQR)	3 (1–7)	5 (2–11)	7 (3–14)	7 (3–14)
In-hospital Mortality % (N)	1.5% (*n* = 553)	4.3% (*n* = 2,745)	7.6% (*n* = 2,827)	11.8% (*n* = 2,750)
Admitted from ED to IP % (N)	54.3% (*n* = 19,387)	74.1% (*n* = 46,377)	79.5% (*n* = 29,478)	76.7% (*n* = 17,758)
Discharge to a destination other than Home. % (N)	5.9% (*n* = 1,063)	15.4% (*n* = 6,443)	29.7% (*n* = 7,589)	35.8% (*n* = 5,132)
Admissions with at least one AEs % (N)	3.0% (*n* = 1,072)	7.7% (*n* = 4,870)	11.4% (*n* = 4,257)	10.6% (*n* = 2,466)

*ED* Emergency Department, *IP* Inpatient, *LOS* Length of Stay, *IQR* Interquartile Range, *AEs* Adverse events

Median ED waiting time increased from 334 min in non-frail patients to 516 min in those with moderate frailty, before decreasing slightly in the severely frail group (460 min). Admission from the ED to IP care increased from 54.3% in non-frail patients to 79.5% in moderate frailty, with a modest decline in severe frailty. IP LOS rose with increasing frailty, from a median of 3 nights in the non-frail to 7 nights in both moderate and severely frail.

In-hospital mortality increased from 1.5% in non-frail admissions to 11.8% in severe frailty, while discharge to non-home destinations rose from 5.9% in non-frail to 35.8% severely frail. Admissions with at least one AE also became more frequent, increasing from 3.0% in non-frail patients to 11.4% in those with moderate frailty, before falling slightly to 10.6% in the severely frail group. The most frequently reported Datix categories contributing to the composite outcome were in-hospital falls, moisture-associated skin damage, pressure ulcers, in-hospital injuries, and discharge or transfer incidents, whereas adverse drug reactions, self-harm, and patient abuse were relatively uncommon (Supplementary Table 2).

### Frailty and risk of AEs

When frailty was modelled in relation to having at least one AE, risk was higher across all the degrees of frailty. Compared to the non-frail patients, those with mild frailty had 2.6 times the risk (RR = 2.59; 95% CI: 2.43–2.76), rising to 3.82 (95% CI: 3.58–4.08) in moderate frailty and 3.55 (95% CI: 3.31–3.80) in severe frailty all *p* < 0.001.

### Interactions between frailty and patient characteristics

#### Age and frailty interaction

**Fig. 1:**
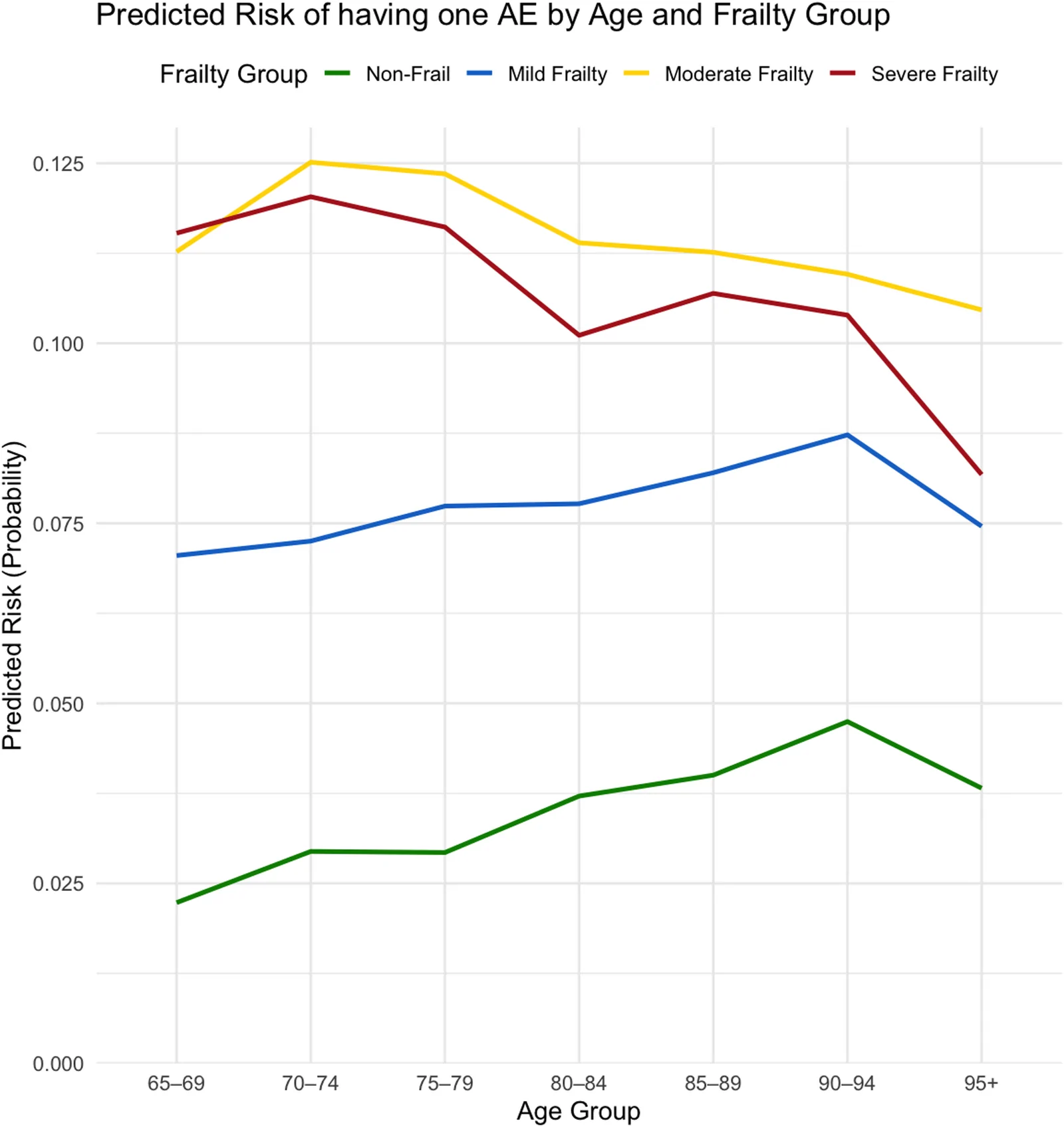
Predicted probability (absolute risk) of experiencing at least one AE by age group and the degrees of frailty, based on the Poisson regression model including their interaction

The interaction between age and frailty was statistically significant in relation to the risk of at least one AE (χ² = 71.11, df = 18, *p* < 0.001), indicating that the association between frailty and AE risk varied across age groups. In patients with no frailty and mild frailty AE risk was higher with increasing age; however, this pattern was not observed in those with moderate and severe frailty, where risk appeared to be lower with age. See Fig. 1.

#### Gender and frailty interaction

The interaction between gender and frailty was not statistically significant (χ² = 0.62, df = 3, *p* = 0.891), indicating similar association between frailty and AE risk in males and females. Given this, we examined gender’s independent association after adjusting for frailty, males showing a 26% higher risk of AE than females (RR = 1.26; 95% CI: 1.22–1.30; *p* < 0.001).

#### Ethnicity and frailty interaction

The interaction between ethnicity and frailty also did not reach statistical significance (χ² = 11.64, df = 15, *p* = 0.706). Accordingly, we examined the association between ethnicity and the risk of having at least one AE after adjusting for frailty, compared with White patients, Asian patients (RR 0.75, 95% CI 0.70–0.79; *p* < 0.001) and lower risks of AEs were observed among Black patients (RR 0.79, 95% CI 0.66–0.95; *p* = 0.01). No significant differences were observed for patients of Mixed ethnicity (RR 1.00, 95% CI 0.67–1.49; *p* = 0.98), other ethnicities (RR 0.85, 95% CI 0.70–1.03; *p* = 0.10), or unknown ethnicities (RR 0.92, 95% CI 0.81–1.05; *p* = 0.22).

#### ED wait time and frailty interaction

**Fig. 2:**
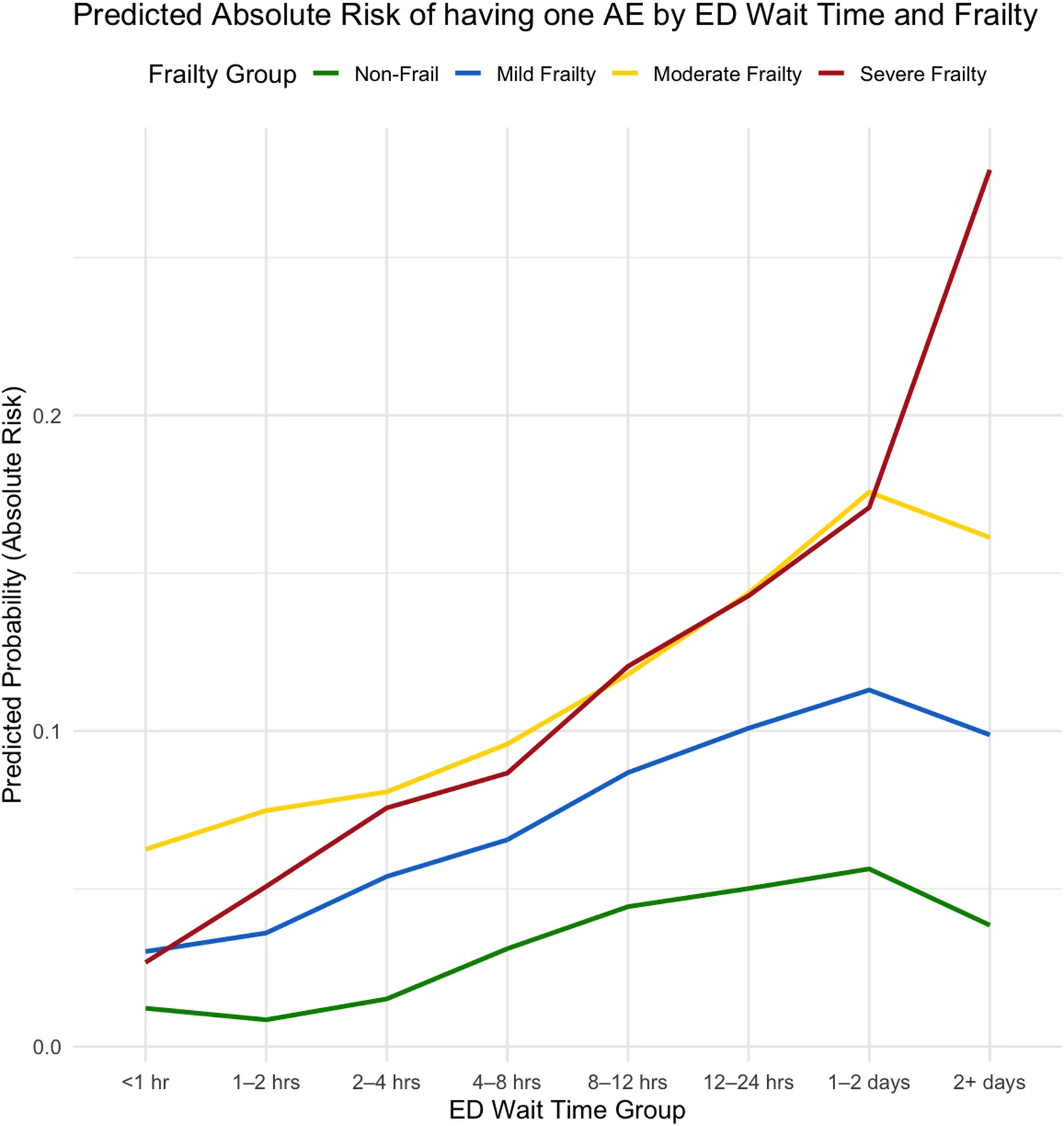
Predicted probability (absolute risk) of experiencing at least one AE by ED wait time group and the degrees of frailty, based on the Poisson regression model including their interaction

The ED wait time and frailty interaction was statistically significant (χ² = 67.25, df = 21, *p* < 0.001). AE risk was higher with longer waits across the degree of frailty, peaking at 1–2 nights in ED; beyond this, the risk appeared to be lower for all degrees of frailty, except in the severely frail, whose risk remained higher. See Fig. 2.

#### IP LOS and frailty interaction

**Fig. 3:**
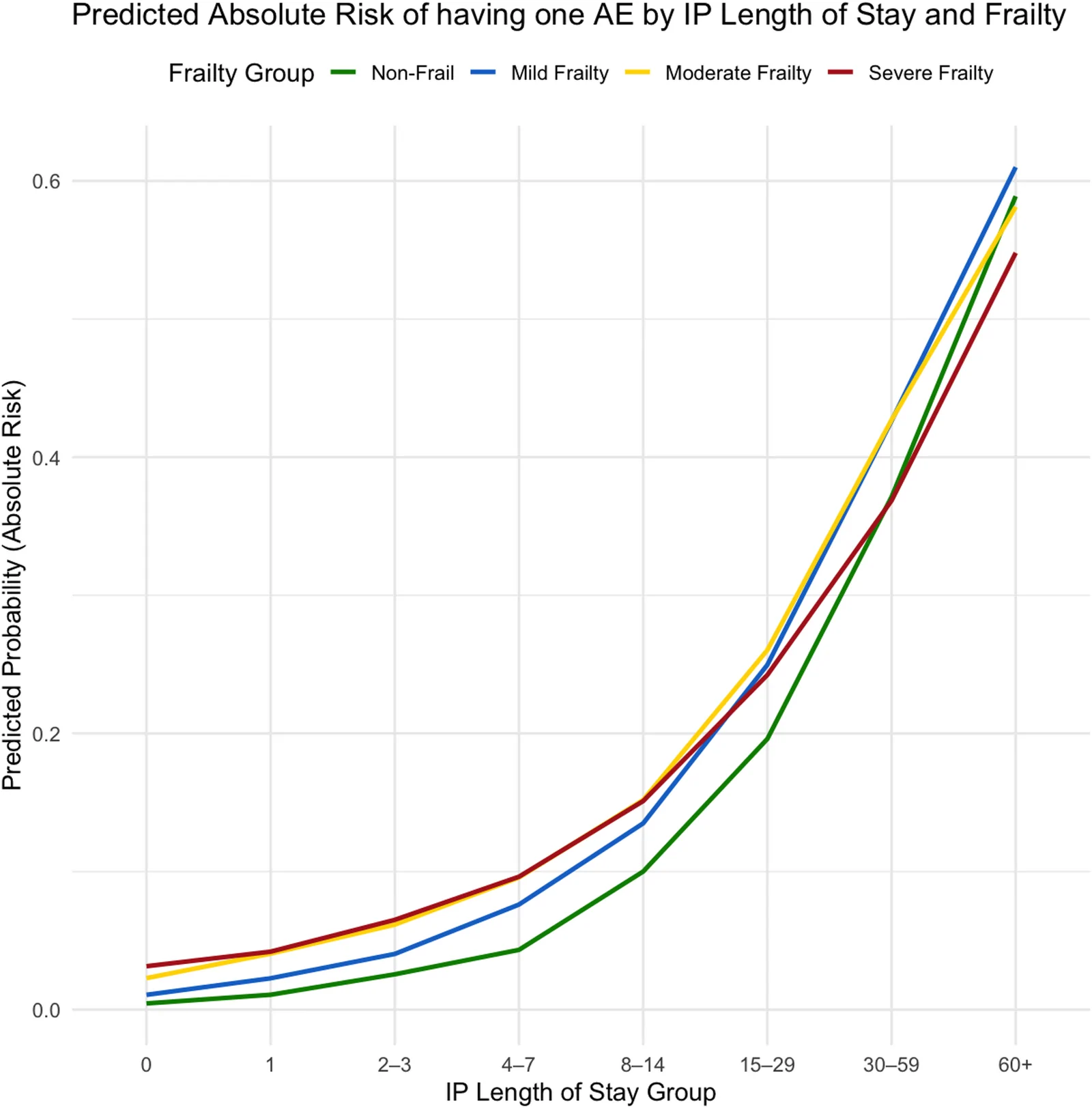
Predicted probability (absolute risk) of experiencing at least one AE by IP LOS group and the degrees of frailty, based on the Poisson regression model including their interaction

The interaction between IP LOS and frailty was statistically significant (χ² = 150.53, df = 21, *p* < 0.001), indicating that the association between IP LOS and AE risk varied by frailty level. The risk was higher across all degrees of frailty. Moderate and severe frailty were associated with higher risk at shorter stays compared to non-frail and mildly frail, but this difference was less apparent in longer IP stays. See Fig. 3.

#### Multivariable interaction model

Starting with a model including all main effects and all interactions, stepwise elimination showed that dropping interactions with age, gender, ethnicity, and ED wait time together with the main effect of age did not statistically significantly affect the model fit. Only the IP LOS and frailty interaction remained significant, with its removal associated with substantial change in the model fit (χ² = 151.32, df = 21, *p* < 0.001). RRs from the multivariable model, including the interaction between IP LOS and frailty, are presented in Supplementary Table 3. The sensitivity analysis using patient-level clustered robust standard errors produced materially similar estimates and did not change the overall conclusions. Collinearity diagnostics showed no evidence of problematic multicollinearity among the main predictors, with VIFs for frailty and IP LOS close to 1.

## Discussion

To our knowledge, this is the first study to examine how multiple patient characteristics interact with the degree of frailty in relation to the risk of experiencing at least one hospital-related AE in older acutely admitted patients. Frailty was independently associated with AEs in older adults acutely admitted through the ED. This association was significantly modified by patient age, ED wait time, and IP LOS. When all patient demographic and clinical characteristics were included in a multivariable model with frailty, removing the frailty–IP LOS interaction was associated with a substantial change in model fit, highlighting its role in explaining variation in AE risk, consistent with existing evidence linking prolonged hospital stay with an increased risk of hospital-associated adverse outcomes in frail patients [19]. Although some patients contributed more than one admission, each admission was analysed as a separate hospital episode. Sensitivity analyses accounting for within-patient correlation produced materially similar findings.

Consistent with our findings, a study of approximately 68,000 CFS assessments reported that frailty documentation was not uniform and varied according to patients’ clinical and demographic characteristics [20]. This suggests that the findings are most applicable to older and clinically complex patients with a documented CFS rather than to all acutely admitted older adults.

The age and frailty significant interaction suggests that the association between frailty and AE risk varies by age. At younger ages, absolute risk was higher with increasing age for the non-frail and mildly frail. In contrast, although moderately and severely frail patients had the highest risk at younger ages, their risk appeared to be lower with advancing age. Although it may sound unexpected, the risk was lower among those aged ≥ 95 years across all degrees of frailty. Few frailty studies have stratified results by age; however, this reduction in the association between frailty and AE risk at extreme older age could be explained by reduced predictive validity of the CFS at extreme ages, where most individuals already have significant deficits, making differences in risk less clear [21]. Additionally, people at extreme age may receive more individualised palliative or restricted care due to their advanced age, which could influence the relationship between frailty and AE risk [21]. This pattern may also reflect survivor bias and competing risks, whereby admitted patients with advanced age represent a highly selected group, resulting in lower observed in-hospital mortality rates compared with younger older adults [22]. Similarly, higher mortality among severely frail patients may have reduced the time at risk for AEs, potentially influencing the observed associations.

The significant interaction between ED wait time and frailty indicates that the association between ED wait time and AEs risk differs across frailty strata. Absolute AE risk was higher with longer waits in all frailty groups, peaking around 1–2 days; beyond this, risk appeared to decline in non‑frail and mildly/moderately frail patients, but remained higher in those with severe frailty. This pattern may reflect the selection of higher‑risk patients to earlier admission, transfer or mortality [23], limited observation at very long waits [24], and the disproportionate vulnerability of severely frail patients to ED boarding‑related harms such as immobility, missed or delayed medications, sleep disruption and missed nursing care [24, 25]. 

The significant interactions between IP LOS and frailty indicates that the association between LOS and AE risk varies by the degree of frailty. At short stays, AE risk was clearly stratified by frailty. As LOS increased, absolute risk was higher across all groups, and between-group differences narrowed; by ≥ 60 days, risks converged to similarly high levels across all degrees of frailty. This pattern suggests that prolonged hospitalisation may play an important role in AE risk. Consistent with this, LOS was the only factor whose removal was associated with a substantial change in multivariable model fit. Potential explanation includes longer time at risk for hospital-acquired complications, cumulative exposure to procedures and invasive devices, and reverse causation, in which AEs extend LOS [26, 27]. Similar associations between extended hospitalisation and poor outcomes in older IP living with frailty have been reported in other studies aligning with our results [19, 28]. 

The lack of significance in frailty-ethnicity may be partially explained by the limited representation of non-white ethnicities, as more than 80% of our cohort were of White background. A previous study conducted in the same population similarly reported higher admission rates among White patients [29]. This finding may reflect persistent barriers to hospital care among South Asian populations, cultural preferences for family-based care of older adults, or issues related to language preferences and financial situation which may also help explain the admission patterns observed in our cohort [30]. 

The lack of significant interaction between gender and frailty is not a new observation; a previous study by Hessey et al. reported the same, finding no significant interaction between gender and frailty in relation to in-hospital mortality and ICU organ support [31]. 

The higher risk of AEs observed in frail individuals may be related to reduced physiological resilience and greater clinical complexity, including polypharmacy, cognitive impairment, and functional decline [8]. Recognising the combined association of frailty and prolonged IP LOS on AE risk highlights the importance of early frailty identification, closer monitoring, and proactive discharge planning. These findings suggest that current care models may not fully meet the needs of frail patients with prolonged hospital stays.

### Limitations

This study has several limitations. As a retrospective observational study, causal relationships cannot be established. Frailty was assessed at a single time point during ED presentation, and changes in frailty status during hospitalisation could not be captured. In addition, frailty assessment was conducted as part of routine clinical practice, which may introduce variability in CFS scoring, particularly at the extremes of frailty where discrimination may be reduced. Only about half of eligible admissions had a recorded CFS score, and as frailty was more often documented in older and clinically complex patients, selection bias cannot be fully excluded. Therefore, the findings may be less generalisable to all acutely admitted older adults.

Some important confounding factors, such as comorbidity burden and medication use, were not available in the dataset and could not be accounted for. Although categorising ED wait time and IP LOS improved clinical interpretability, it may also have reduced precision and influenced the observed interaction patterns. In addition, IP LOS may serve as both a marker of exposure and a consequence of AEs, potentially introducing reverse causation and influencing the observed associations.

The use of the Datix reporting system also introduces limitations, including potential underreporting and variability in reporting practices across clinicians, which may affect data completeness and accuracy. Furthermore, not all recorded incidents necessarily correspond to AEs as defined in the literature, as the system captures a broad range of patient safety incidents, and some clinically relevant AEs (e.g., delirium) may not be consistently recorded. The composite outcome therefore includes incidents with different clinical mechanisms, which may obscure differences between specific incident types. In addition, the outcome did not account for differences in the number or severity of incidents experienced by individual patients, while detailed information on incident severity was not available in the extracted Datix data. Finally, this study was conducted in a single centre, which may limit the generalisability of the findings.

## Conclusion

In conclusion, this study found that the degree of frailty was independently associated with the risk of having at least one AEs during an acute hospital admission in older patients. The association between frailty and AE risk varied according to age, ED wait time and IP LOS. The interaction between frailty and IP LOS showed the strongest association with the risk of AE.

## Supplementary Information


Supplementary Material 1.


## Data Availability

The datasets analysed during the current study are not publicly available due to institutional data governance restrictions.

## Abbreviations

AE: Adverse event
CFS: Clinical Frailty Scale
ED: Emergency department
EHR: Electronic health record
IP: Inpatient
LOS: Length of stay
IQR: Interquartile range
RR: Relative risk
CI: Confidence interval
df: Degree of freedom
WHO: World Health Organisation
