# Supplementary material for: The interaction between frailty and patient characteristics and its association with hospital-related adverse events in older adults
Source: BMC Geriatr. 2026 Jun 23;26:1102. doi: 10.1186/s12877-026-07854-4 (PMC13523424; doi:10.1186/s12877-026-07854-4)
Supplement: Supplementary file 1 [file 12877_2026_7854_MOESM1_ESM.docx]

Appendix

*Supplementary Table 1 Grouping of AEs by types and its associated Datix descriptors.*

| AE Types | Included event as reported in DATIX |
| --- | --- |
| Medication Errors | - Medicine not administered  - Dose or strength was wrong or unclear  - Dispensing of medication issue  - Wrong drug / medicine  - Wrong quantity  - Mismatch between patient and medicine  - Formulation of medication was wrong  - Contra-indication to the use of the medication  - Medication prescribed to which p. had a known allergy  - Expiry date wrong, omitted or passed  - Wrong route for administration of medication  − 10 Times Medication Error  - Wrong / transposed / omitted medicine label  - Omitted medicine or ingredient  - Controlled drug balance discrepancy |
| Adverse Drug Reaction | - Adverse reaction when drug used as intended |
| Falls | - Fall on level ground  - Fall from a height, bed or chair  - Suspected fall  - Fall resulting in head injury  - Fall resulting in fracture |
| Moisture Associated Skin Damage | - Moisture Associated Skin Damage (MASD) |
| Pressure Ulcers | - Hospital Acquired Pressure Ulcer (Awaiting validation)  - Hospital acquired pressure ulcer Validated - No lapses in Care  - Hospital acquired pressure ulcer Validated - Lapses in Care |
| Infections | - Hospital acquired infection  - Wound or Surgical Site Infection  - Influenza (Suspected or Confirmed)  - Clostridium Difficile Toxins (C. Diff) related death  - Norovirus (Suspected or Confirmed)  - Methicillin Resistant Staphylococcus Aureus (MRSA) Bacteraemia  - Invasive Device related infection  - Hazardous and avoidable exposure to infection |
| Patient Abuse | - Physical abuse, assault or violence by staff  - Sexual Abuse by Staff  - Verbal abuse or disruption by staff  - Assault with a weapon by another patient  - Physical abuse, assault or violence by another patient  - Verbal abuse or disruption by another patient |
| Self-Harm | - Self harm  - Attempted suicide, whether proven or suspected |
| Discharge Transfer | - Inappropriate transfer of patient with Dementia/suspected Dementia  - Absconder / missing patient  - Discharge - inappropriate  - Extended stay / episode of care  - Patient discharged with complications  - Unexpected re-admission or re-attendance  - Unplanned admission / transfer to specialist care unit  - Unplanned return to theatre |
| Hospital Injuries | - Trauma related injury (Not Pressure Ulcer)  - Fainting episode/other Medical Cause  - Bed Rails related injury  - Unintended injury in the course of an operation or clinical task  - Injury caused by medical device  - Collision with an object (Not a fall)  - Collision with an object  - Inappropriate patient handling / positioning  - Infusion injury (extravasation)  - Tripped over an object  - Deep Tissue Injury - No lapses in Care  - Deep Tissue Injury - Lapses in Care |

*Supplementary Table 2 N of Admission With at Least One Adverse Event by Each Type*

| Adverse Event Types | CFS 1–3 | CFS 4–5 | CFS 6 | CFS 7–9 |
| --- | --- | --- | --- | --- |
| In-hospital Falls | 422 | 2,032 | 1,586 | 636 |
| Pressure Ulcers | 130 | 559 | 546 | 421 |
| Moisture Associated Skin Damage | 226 | 1,326 | 1,398 | 868 |
| Adverse Drug Reaction | 1 | 11 | 8 | 3 |
| Medication Errors | 111 | 347 | 219 | 153 |
| Hospital-Acquired Infections | 42 | 199 | 219 | 105 |
| Patient Abuse | 7 | 44 | 21 | 15 |
| Self-Harm | 1 | 5 | 5 | 1 |
| Discharge & Transfer | 114 | 380 | 349 | 261 |
| In-Hospital Injuries | 120 | 478 | 368 | 233 |

*Supplementary Table 3 RR of experiencing at least one AE by age, gender, ethnicity, ED wait time, frailty × IP LOS interaction with interaction terms. Non-frail, Female, White Ethnicity, < 1 HR ED wait time and 0-night IP LOS was used as a reference group.*

| Term | RR | CI Lower | CI Upper | P-value |
| --- | --- | --- | --- | --- |
| Intercept | ref | ref | ref |  |
| 70–74 years | 1.075 | 1.002 | 1.153 | 0.043 |
| 75–79 years | 1.061 | 0.992 | 1.135 | 0.086 |
| 80–84 years | 1.055 | 0.987 | 1.127 | 0.118 |
| 85–89 years | 1.068 | 0.999 | 1.141 | 0.054 |
| 90–94 years | 1.067 | 0.992 | 1.147 | 0.081 |
| 95 + years | 1.020 | 0.920 | 1.129 | 0.71 |
| Unknown | 1.010 | 0.891 | 1.146 | 0.874 |
| Asian | 0.826 | 0.782 | 0.873 | < 0.001 |
| Black | 0.822 | 0.694 | 0.974 | 0.024 |
| Mixed | 0.986 | 0.669 | 1.454 | 0.944 |
| Other | 0.850 | 0.707 | 1.023 | 0.086 |
| 1–2 hrs | 0.830 | 0.570 | 1.209 | 0.332 |
| 2–4 hrs | 0.877 | 0.620 | 1.241 | 0.459 |
| 4–8 hrs | 0.914 | 0.647 | 1.290 | 0.608 |
| 8–12 hrs | 1.009 | 0.714 | 1.425 | 0.961 |
| 12–24 hrs | 1.060 | 0.751 | 1.497 | 0.739 |
| 1–2 days | 1.095 | 0.771 | 1.553 | 0.613 |
| 2 + days | 1.405 | 0.832 | 2.375 | 0.204 |
| Mildly Frail (CFS 4–5) | 2.348 | 1.200 | 4.594 | 0.013 |
| Moderately Frail (CFS 6) | 4.958 | 2.493 | 9.861 | < 0.001 |
| Severely Frail (CFS 7–9) | 7.024 | 3.442 | 14.336 | < 0.001 |
| 1 night | 2.401 | 1.219 | 4.729 | 0.011 |
| 2–3 night | 5.647 | 3.032 | 10.517 | < 0.001 |
| 4–7 night | 9.574 | 5.223 | 17.550 | < 0.001 |
| 8–14 night | 22.160 | 12.162 | 40.379 | < 0.001 |
| 15–29 night | 43.265 | 23.735 | 78.865 | < 0.001 |
| 30–59 night | 81.155 | 44.267 | 148.781 | < 0.001 |
| 60 + night | 129.435 | 69.935 | 239.555 | < 0.001 |
| Male | 1.215 | 1.176 | 1.254 | < 0.001 |
| Mildly Frail (CFS 4–5): 1 night | 0.886 | 0.410 | 1.914 | 0.758 |
| Moderately Frail (CFS 6): 1 night | 0.757 | 0.344 | 1.665 | 0.489 |
| Severely Frail (CFS 7–9):1 night | 0.560 | 0.246 | 1.276 | 0.168 |
| Mildly Frail (CFS 4–5): 2–3 night | 0.668 | 0.329 | 1.358 | 0.265 |
| Moderately Frail (CFS 6): 2–3 night | 0.485 | 0.235 | 1.003 | 0.051 |
| Severely Frail (CFS 7–9): 2–3 night | 0.368 | 0.173 | 0.785 | 0.01 |
| Mildly Frail (CFS 4–5): 4–7 night | 0.742 | 0.373 | 1.478 | 0.396 |
| Moderately Frail (CFS 6): 4–7 night | 0.445 | 0.220 | 0.901 | 0.024 |
| Severely Frail (CFS 7–9): 4–7 night | 0.319 | 0.153 | 0.665 | 0.002 |
| Mildly Frail (CFS 4–5): 8–14 night | 0.569 | 0.287 | 1.125 | 0.105 |
| Moderately Frail (CFS 6): 8–14 night | 0.303 | 0.151 | 0.610 | < 0.001 |
| Severely Frail (CFS 7–9): 8–14 night | 0.217 | 0.105 | 0.448 | < 0.001 |
| Mildly Frail (CFS 4–5): 15–29 night | 0.535 | 0.270 | 1.058 | 0.072 |
| Moderately Frail (CFS 6): 15–29 night | 0.266 | 0.132 | 0.534 | < 0.001 |
| Severely Frail (CFS 7–9): 15–29 night | 0.177 | 0.086 | 0.366 | < 0.001 |
| Mildly Frail (CFS 4–5): 30–59 night | 0.479 | 0.241 | 0.954 | 0.036 |
| Moderately Frail (CFS 6): 30–59 night | 0.230 | 0.114 | 0.465 | < 0.001 |
| Severely Frail (CFS 7–9): 30–59 night | 0.142 | 0.068 | 0.295 | < 0.001 |
| Mildly Frail (CFS 4–5): 60 + night | 0.431 | 0.214 | 0.870 | 0.019 |
| Moderately Frail (CFS 6): 60 + night | 0.196 | 0.095 | 0.402 | < 0.001 |
| Severely Frail (CFS 7–9): 60 + night | 0.133 | 0.062 | 0.285 | < 0.001 |
